# FcγRI expression on macrophages is required for antibody-mediated tumor protection by cytomegalovirus-based vaccines

**DOI:** 10.18632/oncotarget.25630

**Published:** 2018-06-29

**Authors:** Hreinn Benonisson, Heng Sheng Sow, Cor Breukel, Jill W.C. Claassens, Conny Brouwers, Margot M. Linssen, Anke Redeker, Marieke F. Fransen, Thorbald van Hall, Ferry Ossendorp, Ramon Arens, Sjef Verbeek

**Affiliations:** ^1^ Department of Human Genetics, Leiden University Medical Center, Leiden, The Netherlands; ^2^ Department of Immunohematology and Blood Transfusion, Leiden University Medical Center, Leiden, The Netherlands; ^3^ Department of Medical Oncology, Leiden University Medical Center, Leiden, The Netherlands

**Keywords:** melanoma, Fc receptors, antibody, cytomegalovirus, vaccines

## Abstract

Cytomegalovirus (CMV)-based vaccine vectors are promising vaccine platforms because they induce strong and long-lasting immune responses. Recently it has been shown that vaccination with a mouse CMV (MCMV) vector expressing the melanoma-specific antigen TRP2 (MCMV-TRP2) protects mice against outgrowth of TRP2-positive B16 melanoma tumors, and this protection was dependent on the induction of IgG antibodies. Here we demonstrate that, although mice lacking all receptors for the Fc part of IgG (FcγRs) develop normal IgG responses after MCMV-TRP2 vaccination, the protection against B16 melanoma was completely abrogated, indicating that FcγRs are indispensable in the downstream effector pathway of the polyclonal anti-TRP2 antibody response. By investigating compound FcγR-deficient mouse strains and by using immune cell type-specific cell ablation we show that the IgG antibody-mediated tumor protection elicited by MCMV-TRP2 mainly depends on FcγRI expression on macrophages, whereas FcγRIV plays only a modest role. Thus, tumor-specific antibody therapy might benefit from combination therapy that recruits FcγRI-expressing pro-inflammatory macrophages to the tumor micro-environment.

## INTRODUCTION

Most vaccines against cancer exhibit less impressive results in comparison with the clinical results of immunomodulatory antibodies and adoptive T cell therapy (ACT) [1]. This could be attributed due to various immune evasion mechanisms and to the failure of inducing long-lasting sustainable functional T and B cell responses. With respect to the latter, cytomegalovirus (CMV)-based vaccines hold great potential because of the ability of CMV to elicit large adaptive immune responses that are maintained without contracting [2]. This phenomenon, termed memory inflation, is observed for the increase in effector-memory T cell populations [3] as well as for IgG antibodies [4]. Other properties such as the ability of CMV to re-infect despite pre-existing immunity [5, 6] and the adaptability of CMV for genetic engineering [7], thereby even allowing large insertions of foreign DNA without losing viral replication potential, also contribute to the value of CMV as a vaccine vector.

The efficacy of CMV as a cancer vaccine vector has been demonstrated in several preclinical models by using MCMV vectors containing prostate specific antigen (PSA), melanoma antigens (gp100, tyrosinase-related protein-2 (TRP-2)) or model antigens (ovalbumin) [8–11]. While the efficacy of most of these MCMV vaccine vectors was centred on the induction of large effector-memory T cell responses, the effectiveness of the MCMV-TRP2 vector was antibody dependent [10], but the mechanisms of actions of the TRP2 antibody-mediated tumor protection remain to be determined.

The B16 transplantable melanoma model is widely used to study underlying mechanisms of tumor-specific antibody-based therapy [12–19]. In most studies the IgG2c (the equivalent of IgG2a in C57BL/6 mice) monoclonal antibody TA99 [12–16, 19] or other IgG subclasses engineered from it [17, 18] specific for the B16-F10 antigen TRP1 (gp75) were used. In addition, active immunisation with gp75 protein was applied [15]. In contrast to anti-HER2/Neu antibody in breast cancer, TA99 does not interfere with cell signalling when bound to its tumor target and has no direct effect on growth or survival of tumor cells. Therefore, the therapeutic efficacy of TA99 depends on its ability to recruit effector cells of the immune system, which can kill the tumor cells by different mechanisms including antibody dependent cell cytotoxicity (ADCC), antibody dependent cell phagocytosis (ADCP), trogocytosis [20], complement dependent cell-mediated phagocytosis (CDCP), and cell-mediated cytotoxicity (CDCC) [21].

Four different FcγRs have been identified in the mouse. The IgG binding α-chains of the activating FcγRI, FcγRIII and FcγRIV are associated with the FcR γ chain, a signal transduction subunit that is also required for cell surface expression. The activating FcγR are counterbalanced by the inhibiting receptor FcγRIIb. The four FcγRs are expressed in different combinations on a variety of immune cells, mainly myeloid effector cells [22, 23]. Different laboratories reported contradictory results regarding the involvement of the individual activating FcγRs using one of the three B16-F10 tumor model variants and different panels of FcγR deficient mice and FcγR blocking antibodies [13, 14, 17–19].

Here we aim to decipher the underlying mechanisms of the anti-tumor effects of TRP2 polyclonal antibodies elicited by MCMV-TRP2 vaccination through investigating the individual role of the activating FcγRI, FcγRIII and FcγRIV and the different FcγR-expressing immune effector cells. We demonstrate that after immunization with the MCMV-TRP2 vector, the protection against tumor outgrowth mediated by the elicited polyclonal TRP2 antibodies is completely abrogated in FcγRI-deficient mice and partially diminished in FcγRIV^−/−^ mice. Cell subset depletion revealed that macrophages were the main innate effector immune cells involved.

## RESULTS

### Germinal center reactions and antibody responses during CMV infection are not FcγR dependent

To investigate the particular role of FcγRs in the antibody-mediated protection induced by MCMV-TRP2 vectors [10], we initially aimed to investigate whether FcγRs impact B cell responses including the development of germinal center (GC) B cells and plasma cells, T follicular helper (T_FH_) cell responses, and antibody responses upon CMV infection.

**Figure 1:**
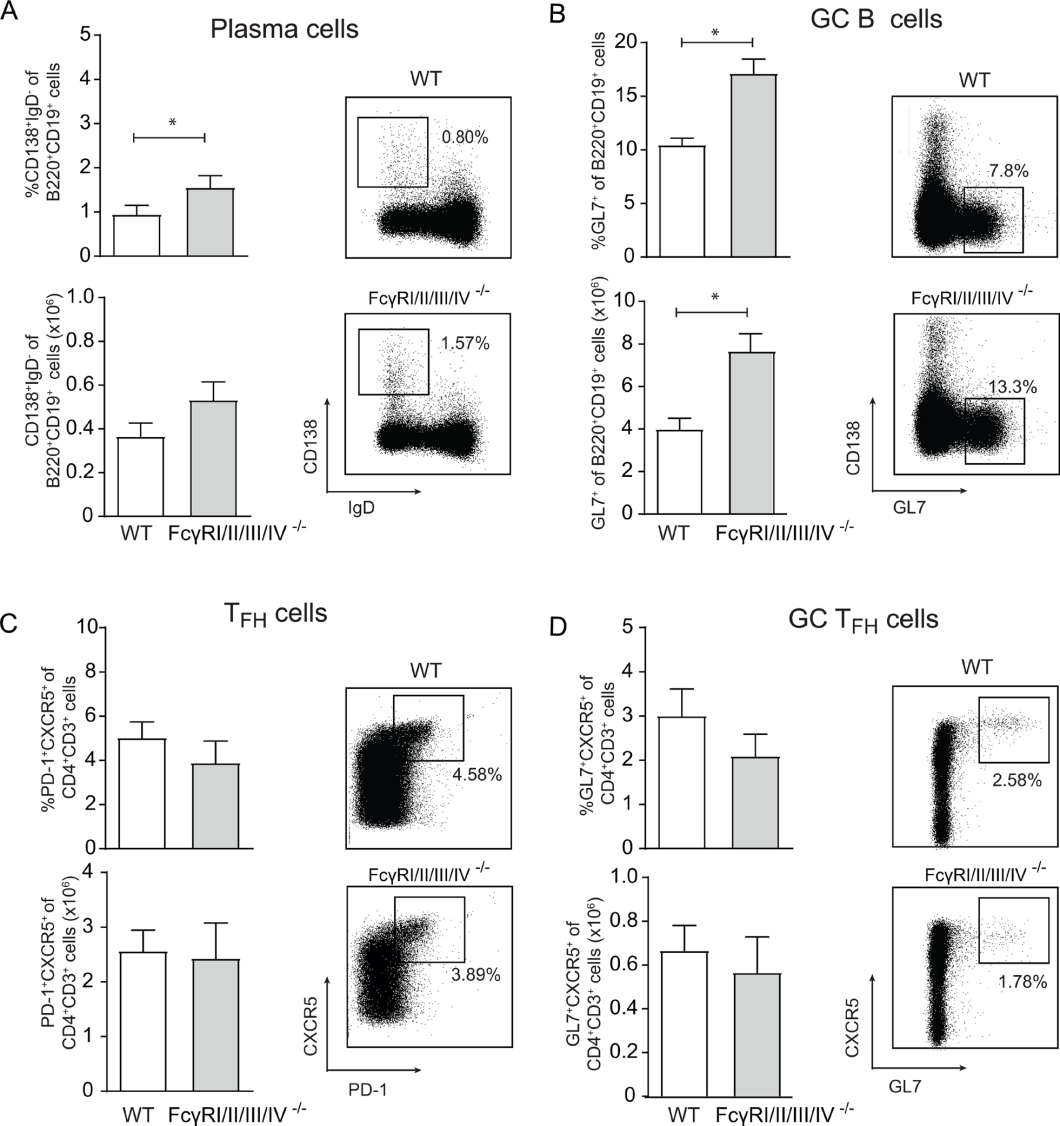
Germinal center reactions upon CMV infection are not abrogated in mice lacking all FcγRs (**A**–**D**) WT and FcγRI/II/III/IV^−/−^ mice were infected with MCMV-Smith and at day 14 post-infection the B cell and T cell germinal center (GC) responses in the spleen were examined. Representative flow cytometry plots are shown. (A–B) Percentage and absolute number of plasma cells (CD138^+^IgD^–^) and GC B cells (GL7^+^) within splenic CD19^+^ B220^+^ B cells in WT and FcγRI/II/III/IV^−/−^ mice. (C–D) Percentage and total cell numbers of (C) T_FH_ (CXCR5^+^PD1^+^) cells and (D) GC T_FH_ (CXCR5^+^GL7^+^) cells within CD4^+^CD3^+^ T cells are shown. Results from one representative experiment out of three performed are shown (^*^p < 0.05, *n* = 5).

**Figure 2:**
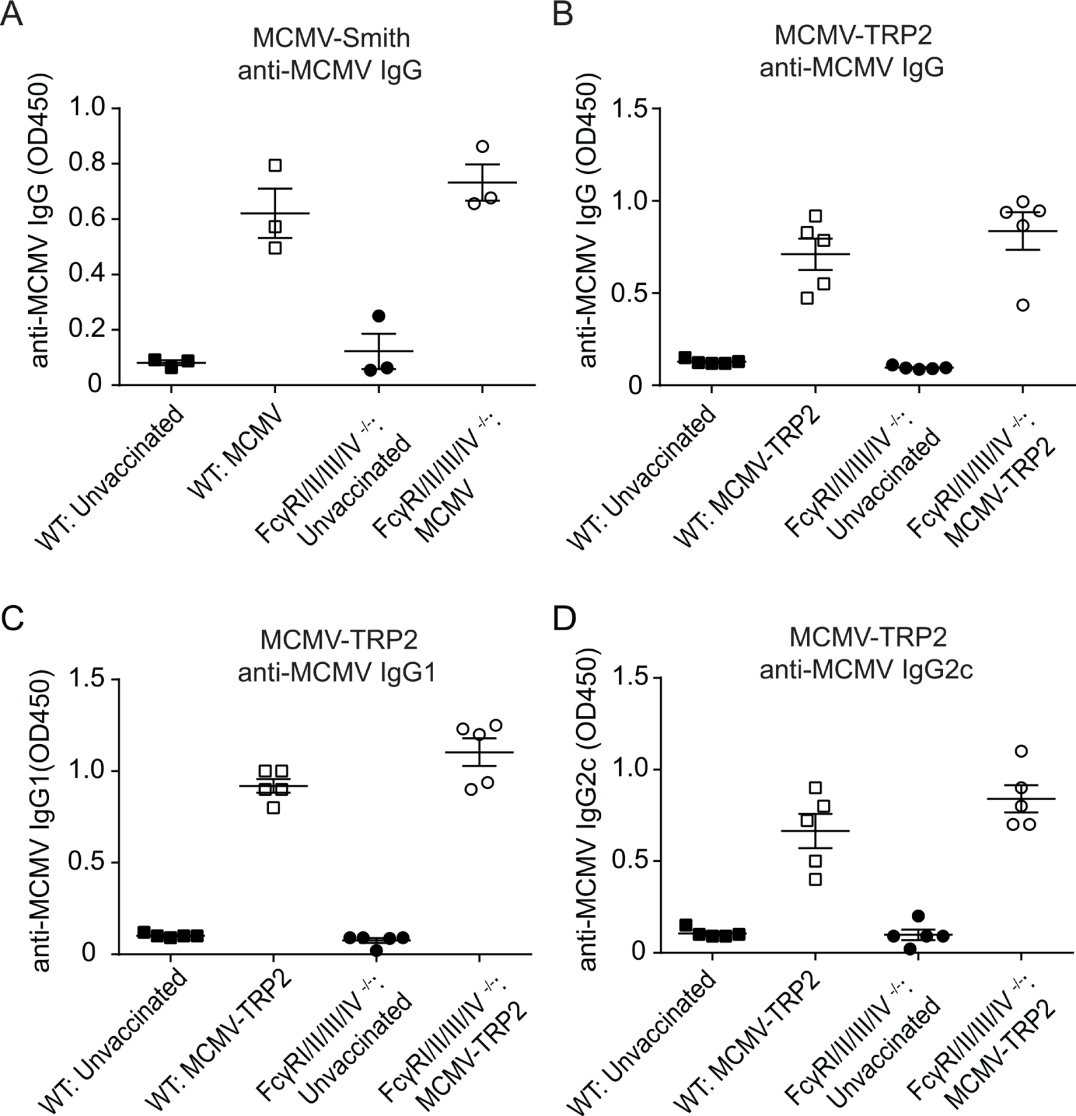
FcγR deficiency does not alter the anti-MCMV antibody response after MCMV-TRP2 vaccination (**A**) Levels of MCMV-specific total IgG in serum of WT and FcγRI/II/III/IV^−/−^ mice at day 14 post-infection with MCMV-Smith. Measurement at OD450 of 1:400 diluted serum. (**B**) Levels of MCMV-specific total IgG in serum of WT and FcγRI/II/III/IV^−/−^ mice at day 14 post vaccination with MCMV-TRP2. Measurement at OD450 of 1:100 diluted serum. (**C**) Levels of MCMV-specific IgG1 in serum of WT and FcγRI/II/III/IV^−/−^ mice at day 14 post vaccination with MCMV-TRP2. Measurement at OD450 of 1:100 diluted serum. (**D**) Levels of MCMV-specific IgG2c in serum of WT and FcγRI/II/III/IV^−/−^ mice at day 14 post vaccination with MCMV-TRP2. Measurement at OD450 of 1:100 diluted serum.

Wild-type (WT) C57BL/6 mice and mice lacking all four FcγRs were infected with MCMV-Smith and analysed at day 14 post-infection. The GC B cell responses, and plasma cells were slightly increased in FcγRI/II/III/IV^−/−^ mice (Figure 1A and 1B) compared to WT mice, while the GC T_FH_ cell responses were comparable (Figure 1C and 1D). The IgG antibody responses specific to MCMV at day 14 post-infection with MCMV-Smith were comparable between WT and FcγRI/II/III/IV^−/−^ mice (Figure 2A). Consistent with natural infection, the IgG antibody responses (total IgG, IgG1, IgG2c) to MCMV after vaccination with MCMV-TRP2 were also similar in WT and FcγRI/II/III/IV^−/−^ mice (Figure 2B–2D). Thus, FcγRs have a minor impact on GC reactions and antibody formation, indicating that potential effects of FcγR-deficiency cannot be attributed to insufficiencies in antibody responses.

### FcγRI is predominantly required for the antibody-mediated tumor protection induced by MCMV-TRP2 vaccination

**Figure 3:**
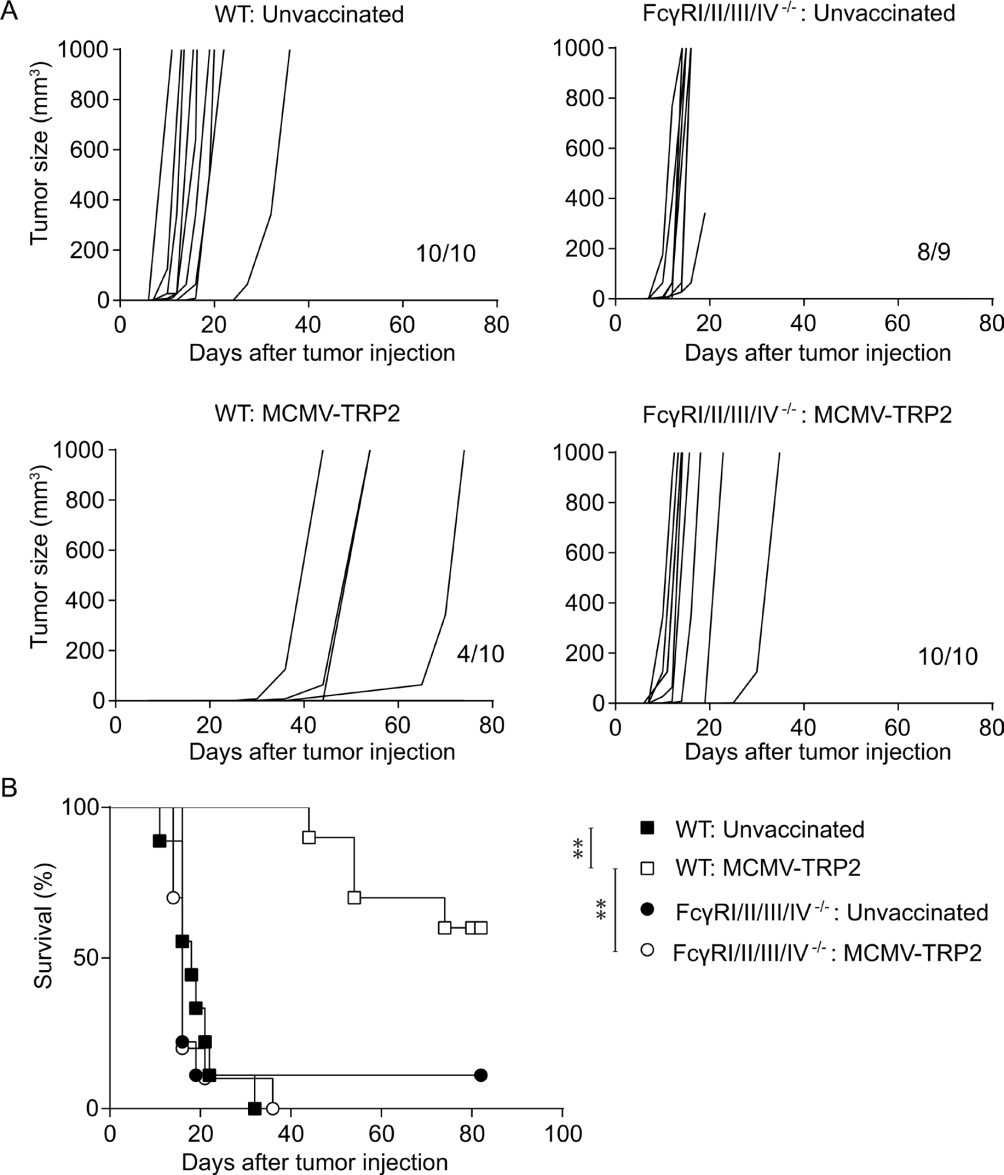
Protection against B16 tumor outgrowth by MCMV-TRP2 vaccination is FcγR dependent WT and FcγRI/II/III/IV^−/−^ mice were vaccinated with MCMV-TRP2 or left unvaccinated, and at day 14 post-vaccination challenged with B16 tumor cells. (**A**) Tumor growth curves of B16 tumor challenged WT and FcγRI/II/III/IV^−/−^ mice following MCMV-TRP2 vaccination. Numbers at the bottom right of each panel represent the number of mice from total that succumbed to the tumor challenge. (**B**) Survival of B16 tumor-bearing WT and FcγRI/II/III/IV^−/−^ mice following vaccination with MCMV-TRP2. Results are shown from two pooled experiments (^*^p < 0.05, ^**^p < 0.01, *n* = 10).

Next, we aimed to analyse whether FcγRs play a role in the IgG-mediated effector mechanisms that are operational during tumor protection upon vaccination with MCMV-TRP2. To determine whether the MCMV-TRP2-induced anti-tumor immunity is FcγR dependent, WT mice and mice deficient in all four FcγRs were vaccinated with MCMV-TRP2, and two weeks later challenged with B16 tumor cells. In untreated WT and FcγRI/II/III/IV^−/−^ mice the outgrowth of B16 tumor was similar. In contrast, MCMV-TRP2 vaccination elicited a clearly delayed onset of B16 tumor outgrowth in WT mice but not in FcγRI/II/III/IV^−/−^ mice (Figure 3). Thus, the antibody-mediated protection induced by MCMV-TRP2 vaccination is FcγR dependent.

**Figure 4:**
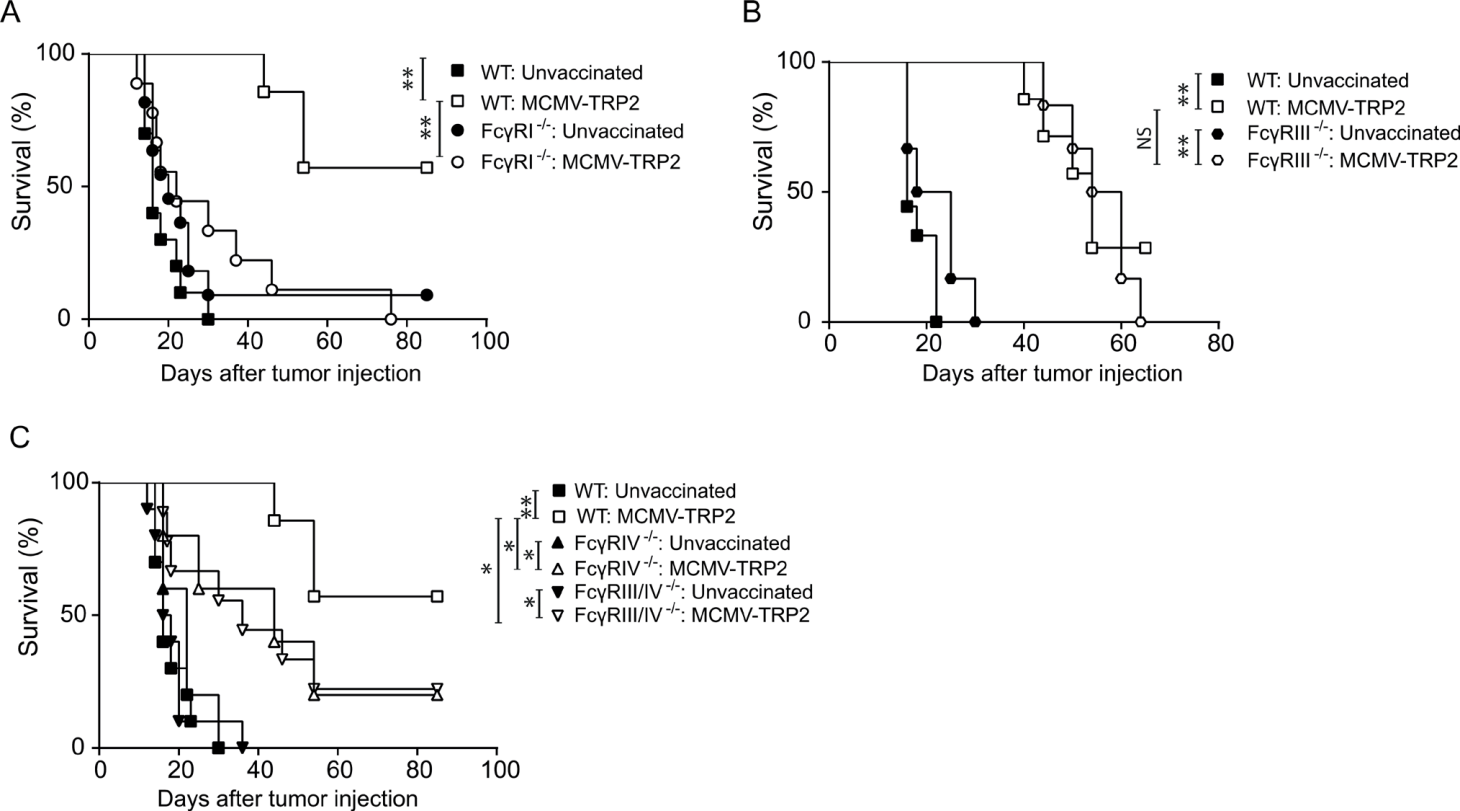
Protection against B16 tumor outgrowth by MCMV-TRP2 vaccination is mainly FcγRI dependent WT mice and mice deficient in one or more FcγRs were vaccinated with MCMV-TRP2 or left unvaccinated, and at day 14 post-vaccination challenged with B16 tumor cells. (**A**) Survival of B16 tumor challenged WT and FcγRI^−/−^ mice. (**B**) Survival of B16 tumor challenged WT and FcγRIII^−/−^ mice. (**C**) Survival of B16 tumor challenged WT, FcγRIV^−/−^ and FcγRIII/IV^−/−^ mice. Results shown are from two pooled experiments (^*^p < 0.05, ^**^p < 0.01 *n* = 5–11).

The expression profiles vary between FcγR classes as well as the downstream antibody effector functions they facilitate. To gain insight in the contribution of the individual activating FcγRs, the protection against outgrowth of the B16 tumor after vaccination with MCMV-TRP2 was analysed in mice deficient for one or two FcγR classes. The generation of FcγRIV^−/−^ and FcγRIII/IV^−/−^ mouse strains is described in the Supplementary Data. FcγRI^−/−^, FcγRIII^−/−^, FcγRIV^−/−^ and FcγRIII/IV^−/−^ mice were vaccinated with MCMV-TRP2 or left unvaccinated, and two weeks later challenged with B16 tumor cells. All unvaccinated WT and FcγR^−/−^ mice developed progressive tumors within 2–3 weeks after challenge. Whereas vaccinated WT and FcγRIII^−/−^ mice showed a substantial increased survival, MCMV-TRP2 vaccinated FcγRI^−/−^ mice did not (Figure 4A and 4B). FcγRIV^−/−^ and FcγRIII/IV^−/−^ mice showed a delay in tumor outgrowth, albeit significantly less compared to MCMV-TRP2 vaccinated WT mice (Figure 4C). Taken together these data suggest that FcγRI plays a dominant role in the antibody-mediated tumor protection induced by MCMV-TRP2 vaccination whereas FcγRIV has moderate impact.

### Tumor protection by MCMV-TRP2 vaccination predominantly requires macrophages

**Figure 5:**
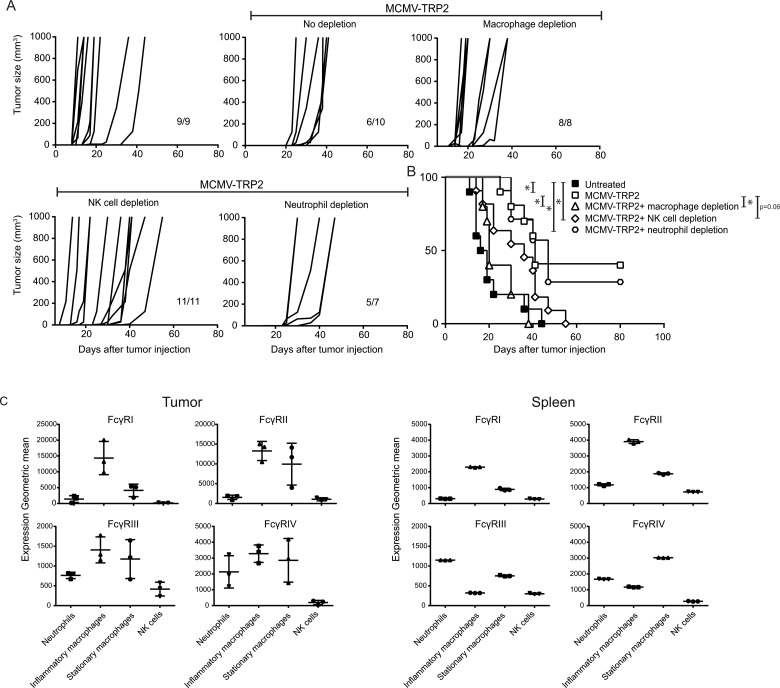
Protection against B16 melanoma outgrowth by MCMV-TRP2 vaccination requires macrophages WT mice were vaccinated with MCMV-TRP2 or left unvaccinated, and at day 14 post-vaccination challenged with B16 tumor cells. Macrophages, NK cells or neutrophils were depleted right before and during tumor challenge. (**A**) Tumor growth curves of B16 tumor challenged WT mice in which the indicated cell subsets have been depleted following vaccination with MCMV-TRP2. Numbers at the bottom right of each panel represent the number of mice from total that survived the treatment. Results are shown from two pooled experiments. (**B**) Survival of B16 tumor-bearing WT mice in which the indicated cell subsets have been depleted following vaccination with MCMV-TRP2. Results are shown from two pooled experiments (^*^p < 0.05, ^**^p < 0.01, *n* = 9–11). (**C**) Expression of FcγRI, FcγRII, FcγRIII and FcγRIV on inflammatory macrophages (F4/80^+^ Ly6C^+^), stationary macrophages (F4/80^+^ Ly6C^−^), neutrophils (Ly6G^+^) and NK cells (NK1.1^+^) in tumor and spleen of unvaccinated mice at day 14 post tumor challenge.

FcγRs are variably expressed by different cell types including phagocytic myeloid cells, neutrophils and NK cells [22, 23]. To determine which FcγR-expressing effector cell types are involved in the antibody mediated tumor protection induced by MCMV-TRP2 vaccination, the outgrowth of B16 cells was monitored after s.c. transplantation, followed two days later by depletion of the following cell types: macrophages (depleted by clodronate liposomes), neutrophils (depleted by anti-Ly6G antibody) and NK cells (depleted by anti-NK1.1 antibody). After the depletion of macrophages, the protection against B16 tumor challenge upon MCMV-TRP2 vaccination was abrogated while depletion of NK cells had a slight effect. In addition, depletion of neutrophils did not alter tumor protection (Figure 5A and 5B).

The dominant importance of macrophages is in line with the expression of the FcγRs on these cells. Notably, FcγRI is highly expressed on inflammatory macrophages (Ly6C^+^ F4/80^+^) residing in the tumor and spleen (Figure 5C). Neutrophils express FcγRIII and FcγRIV, while NK cells only express low levels of FcγRIII. Thus, macrophages expressing high levels of FcγRI are the dominant cell type involved in the tumor protecting effects mediated by polyclonal antibodies elicited via MCMV-TRP2 vaccination.

### Macrophages mediate antibody-dependent killing of tumor cells

**Figure 6:**
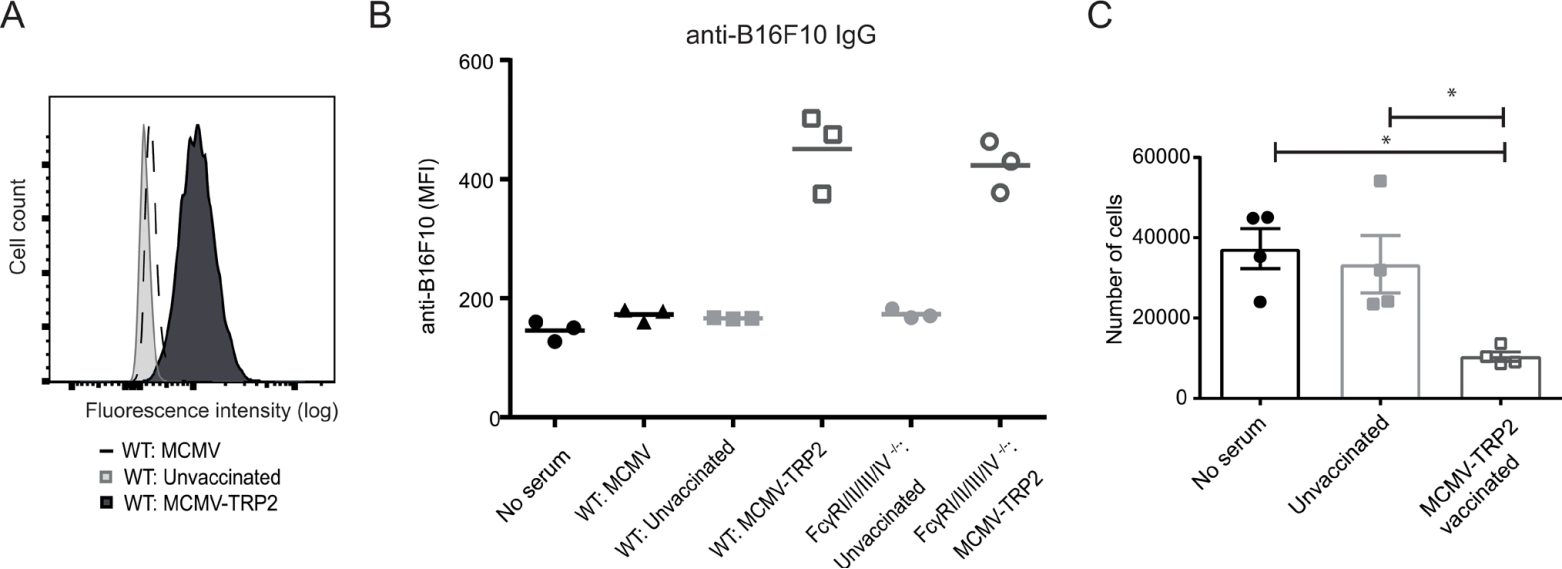
Macrophages mediate antibody-dependent killing of tumor cells (**A**) Histogram showing cell surface staining of B16-F10 tumor cells with 1:100 diluted serum from MCMV infected mice (day 14 post infection), MCMV-TRP2 vaccinated mice (day 14 post-vaccination), and unvaccinated mice. Tumor cells were first incubated with serum and subsequently stained with an Alexa488-conjugated anti-mouse IgG specific antibody. (**B**) B16-F10 tumor-specific staining as described above with no serum and with serum from MCMV infected mice (day 14 post infection), unvaccinated WT and FcγRI/II/III/IV^−/−^ mice, and MCMV-TRP2 vaccinated WT and FcγRI/II/III/IV^−/−^ mice (day 14 after vaccination). (**C**) B16-F10 tumor cells were incubated with macrophages in absence of serum or in presence of serum from non-vaccinated mice and MCMV-TRP2 vaccinated mice (^*^p < 0.05). The number of tumor cells is indicated.

To gain insight into the mechanisms of macrophage-dependent killing of the B16 tumor cells, we first performed cell surface stainings of B16 tumor cells with serum from MCMV-TRP2 vaccinated WT and FcγRI/II/III/IV^−/−^ mice followed by an Alexa488-conjugated anti-mouse IgG specific antibody (Figure 6A and 6B). The cell surface of the B16 tumor cells were positively stained with serum from MCMV-TRP2 vaccinated WT mice while serum from unvaccinated WT mice or serum from WT mice that were infected with MCMV not expressing TRP2 resulted in negative staining. Staining with serum from MCMV-TRP2 vaccinated FcγRI/II/III/IV^−/−^ mice resulted also in a positive cell surface staining whereas serum from unvaccinated FcγRI/II/III/IV^−/−^ mice did not.

To show that macrophages can kill B16F10 cells via serum antibodies that are able to bind to the cell surface of the B16F10 cells, we co-cultured these tumor cells with macrophages (RAW264.7, a murine macrophage cell line) in presence and absence of serum of vaccinated and non-vaccinated mice. We observed that only serum from MCMV-TRP2 vaccinated mice resulted in lysis of the tumor cells (Figure 6C). Thus, macrophages can mediate killing of the B16F10 cells via serum antibodies elicited by the MCMV-TRP2 vaccine, indicating that ADCP is the main effector mechanism involved.

## DISCUSSION

This study shows that a MCMV-based vaccine vector, expressing the full native protein of the melanoma specific antigen TRP2, induced a strong polyclonal antibody response independent of FcγRs. However, these tumor-specific antibodies required FcγRs for their protective effects against the outgrowth of the TRP2 expressing B16 melanoma cells. In particular, FcγRI and to a lower extend FcγRIV expressed on macrophages was required for the tumor protection.

A previous study showed that protection against tumor outgrowth by vaccination with MCMV-TRP2 was not dependent on CD8 or CD4 T cells but rather on antibodies [10]. Other MCMV-based vaccinations in mice have shown to be dependent on the induction of tumor-specific CD8 T cell responses [8, 9, 11, 24]. The most effective anti-tumor CD8 T cell responses were induced with MCMV vectors expressing altered versions or short peptides derived from tumor antigens instead of the full native protein. The MCMV vector used in this study, MCMV-TRP2, expresses the full native TPR2 protein, which might explain the elicitation of antibody responses rather than CD8 T cell responses.

We explored the underlying mechanisms of the therapeutic effect of the polyclonal IgG response induced by MCMV-TRP2. We found that the therapeutic efficacy depended mainly on FcγRI and partially on FcγRIV. Macrophages, expressing high levels of FcγRI and FcγRIV, were in particular crucial for the efficacy of MCMV-TRP2, indicating that FcγRI expression on macrophages is crucial. Most likely crosslinking of the high affinity FcγRI on macrophages induces ADCP of the B16 tumor cells located subcutaneously. Consistent with this are the results of experiments using intravital microscopy in which it was shown that ADCP by macrophages is prominent in the removal of circulating tumor cells through FcγRI and FcγRIV [16]. ADCP may thus be important for cancer vaccines inducing antibody responses and for antibody therapy of cancer. NK cells seem to play a minor role in the anti-tumor protection by the MCMV-TRP2 vaccine. Since NK cells only express FcγRIII and this FcγR is not important in the anti-tumor effects elicited by the MCMV-TRP2 vaccine vector it is expected that the minor anti-tumor effect of NK cells is not mediated via FcγRIII and antibodies.

MCMV-TRP2 induced a polyclonal IgG antibody response of different subclasses that are known to mediate different downstream effector functions. Most tumor-specific antibodies currently used in clinics are monoclonal antibodies. These antibodies have mostly been humanized for their usage in patients. It is known that entirely human antibodies are longer lasting as they have a lower likehood to induce anti-antibody responses [25]. Essentially, vaccination should be able to induce fully human antibodies against the tumor target. Moreover, polyclonal antibodies inititate responses against multiple epitopes thereby minimizing tumor escape. Furthermore, it has been reported that antibodies recognizing multiple epitopes of epidermal growth factor receptor (EGFR) were able to induce complement-dependent tumor cell killing while a monoclonal antibody was not [26]. As such polyclonal antibodies against multiple epitopes could thus also be better at inducing complement-dependent lysis. A mixture of specificities in serum has been shown to induce increased complement activation [27]. Although, in this study we found a promonent role for FcγR-dependent mechanisms, we do not rule out that complement induced lysis could also play a role.

In conclusion our study showed that MCMV-based vaccine vectors can induce strong IgG antibody responses against tumors involving FcγRI expressing macrophages. We anticipate that additional treatments, such as those counter-acting the immunosuppressive tumor micro-environment, could act in synergy in order to improve the therapeutic efficacy of antibodies. Thus, CMV-based vectors are promising anti-cancer vaccines and may become a potent component of an effective combination therapy in cancer.

## MATERIALS AND METHODS

### Mice and antibodies

Mice were housed in the SPF animal facilities of the Central Animal Facility (PDC) of the Leiden University Medical Center (LUMC). The health status of the animals was monitored over time. Animals tested negative for all agents listed in the FELASA (Federation of European Laboratory Animal Science Associations) guidelines for SPF mouse colonies. All mouse studies were approved by the animal ethics committee of the LUMC. Experiments were performed in accordance with the Dutch Act on Animal Experimentation and EU Directive 2010/63/EU (‘On the protection of animals used for scientific purposes’). C57BL/6J mice were purchased from Charles River laboratories. FcγR^−/−^ mice were generated in the transgenic mouse facility of the LUMC (i.e., FcγRI^−/−^ [28], FcγRI/II/III/IV^−/−^ [29]. The generation of FcγRIV^−/−^ and FcγRIII/IV^−/−^ mouse strains is described in the Supplementary Data (Supplementary Figures 1 and 2). The EIIaCre deleter strain (>20 generations backcrossed on a C57BL/6J background), was a kind gift of Dr. Heiner Westphal. The Flp deleter strain C57BL/6-Tg(CAG-flpe)36Ito/ItoRbrc (>20 generations backcrossed on a C57BL/6J background) was purchased from Jackson Laboratories (Bar Harbor, Me). Mice were routinely checked for their genotype by PCR analysis.

### Vaccination, tumor challenge, and cell subset depletion

WT C57BL/6J, FcγRI^−/−^, FcγRIII^−/−^, FcγRIV^−/−^ and FcγRIII/IV^−/−^ mice were intraperitoneally (i.p.) inoculated with 2 × 10^6^ pfu MCMV-TRP2 [10] (kindly provided by Ann Hill, Department of Molecular Microbiology and Immunology, Oregon Health and Science University, Portland, Oregon, USA). Mice were 14 days post vaccination subcutaneously (s.c.) challenged with 2 × 10^5^ B16-F10 melanoma cells in 100 μl PBS. Tumor growth was measured two or three times a week in three dimensions (width, length and height) using a caliper. Mice were euthanized when tumor size reached >2,000 mm^3^ in volume or when mice lost >20% of their total body weight (relative to initial body mass). Mice were bled for serum samples at day 7, 14 and 21 days after infection.

To examine germinal center (GC) responses, WT C57BL/6J and FcγRI/II/III/IV^−/−^ mice were infected with 5 × 10^4^ pfu MCMV-Smith or 2 × 10^6^ pfu MCMV-TRP2 and at day 14 post-infection spleens were harvested and the plasma cells, GC B cells, T follicular helper (T_FH_) cells, GC T_FH_ cells were analyzed by flow cytometry.

For specific cell subset depletion, vaccinated and unvaccinated mice were injected with either 200 μl clodronate liposomes intravenously (Liposoma B.V.), 100 μg anti-NK1.1, or 100 μg anti-Ly6G (1A8) (Bioxcell) intraperitoneally once a week starting two days before tumor inoculation to deplete phagocytic macrophages, NK cells or neutrophils, respectively. Blood samples were analysed by flow cytometry to determine the efficacy of the cell subset depletion.

### Flow cytometry

Blood cells, tumor cells and splenocytes were phenotyped by flow cytometry as described [4, 30]. Briefly, single cell suspensions of spleens were prepared by mincing the spleens through 70-μm cell strainers (BD Biosciences). For FcγR cell surface staining of tumor-infiltrated immune cells, tumors were isolated from mice that were transcardially perfused with PBS-EDTA. Subsequently, tumors were mechanically disrupted in small pieces, incubated for 15 minutes at 37° C in Iscove's modified Dulbecco's medium (IMDM)-containing Liberase (Roche), minced through 70 μm cell strainers and resuspended in staining buffer (PBS + 2% FSC + 0.05% sodium azide) containing antibodies for 30 minutes at 4° C. Erythrocytes were removed by hypotonic lysis.

Fluorochrome-labelled antibodies specific for CD3, CD4, CD8, CD11b, CD95, CD138, GL7, Ly6C, Ly6G, F4/80, FcγRI (CD64), FcγRII (CD32), FcγRIII (CD16), FcγRIV (CD16.2), NK1.1 and IgD, were purchased from BD Biosciences, Biolegend, eBioscience and R&D Systems. Samples were acquired with LSRII flow cytometers (BD Biosciences) and data was analyzed with FlowJo software (TreeStar).

To stain B16-F10 tumor cells with serum, cells were plated in 48 well plates and incubated with serum obtained from MCMV-TRP2 and MCMV vaccinated mice at day 14 post-infection. Subsequently, cells were stained with Alexa488-conjugated goat anti-mouse as a secondary antibody, and subjected to flow cytometry as described above.

### ELISA

MCMV-specific antibodies in serum were detected by ELISA as described [31]. Briefly, 96-well plates (Nunc maxisorp) were coated overnight 4° C with NIH-3T3 derived MCMV-Smith in bicarbonate buffer. Plates were then blocked at 37° C with blocking buffer (5 % milk in PBS) for 1 hour. Sera were diluted in 1% milk PBA and incubated in the blocked wells for 1 hour. HRP conjugated anti-IgG antibodies (Southern biotech, Birmingham, USA) were diluted 1:4000 in 1% milk PBS and incubated 1 hour at 37° C. To develop the plates 100 μl of TMB (sigma Aldrich) was added to each well and incubated for 15 min at room temperature after which 100 μl of stop solution (1 M H_2_SO_4_) was added. Plates were measured using a microplate reader.

### ADCP assay

Macrophages (RAW264.7) were incubated with B16-F10 cells in a 2:1 ratio in 96 well plates. Serum from MCMV-TRP2 vaccinated mice, unvaccinated or no serum was added to the wells. 48 hours later the number of tumor cells were determined by flow cytometry.

### Statistics

Mann–Whitney tests were used to check statistical significance between groups in antibody titers and number of positive cells. Mantel-Cox rank tests were used to check significance between survival of mice between groups from Kaplan Meier plots.

## SUPPLEMENTARY MATERIALS FIGURES


