# Supplementary material for: FcγRI expression on macrophages is required for antibody-mediated tumor protection by cytomegalovirus-based vaccines
Source: Oncotarget. 2018 Jun 29;9(50):29392–402. doi: 10.18632/oncotarget.25630 (PMC6047664; doi:10.18632/oncotarget.25630)
Supplement: Supplementary file 1 [file oncotarget-09-29392-s001.pdf]

FcγRI expression on macrophages is required for antibody-mediated tumor protection by cytomegalovirus-based vaccines

SUPPLEMENTARY MATERIALS

Generation of a novel FcγRIV and FcγRIII/IV$^{-/-}$ mouse models

In the mouse the FcγRIII and FcγRIV genes are located next to each other on chromosome 1. For the generation of the FcγRIII/IV double knock-out (KO) mouse on C57BL/6 background a previously generated Bruce4 ES cell clone with a floxed FcγRIII allele (Fransen MF *et al.*, J Immunol. 2018; 200:2615–26) was targeted with a FcγRIV specific targeting vector that was based on a 11.2 kb genomic fragment containing exon 3, encoding extracellular domain 1 (EC1), exon 4, encoding extracellular domain 2 (EC2), and exon 5, encoding transmembrane and cytoplasmic domain (TM/C) of the FcγRIV gene. This fragment was subcloned by means of *Recombineering* from BAC clone RPCI23-87B18 of the RPCI 23 Female (C57BL/6J) mouse BAC genomic library (BACPAC Resources Center, Children's Hospital Oakland Research Institute, Oakland, California). Recombineering was also used to insert a single LoxP site downstream of the EC2 exon as well as a LoxP-FRT-NEO-FRT cassette upstream of the EC2 exon (Supplementary Figure 1). Clones in which homologous recombination occurred were identified by Southern blotting (not shown) and subsequently injected in C57Bl/6 blastocysts. Recombinant ES cell clones targeted in trans where used to generate a single FcγRIV conditional KO strain (Supplementary Figure 1A) whereas the ES cell clones targeted in sis where used to generate a conditional FcγRIII/IV double KO strain. The obtained chimeras were crossed with WT C57Bl/6J mice and the F1 offspring positive for the FcγRIV targeted allele was crossed with a Flp deleter strain to remove the selection marker cassette, resulting in mice with a floxed FcγRIV allele. Flp-mediated recombination was analyzed with PCR (Supplementary Figure 1B). Crossing these mice with the C57BL/6J EIIACre deleter strain resulted in a full FcγRIV deficiency (FcγRIV$^{-/-}$) and a full FcγRIII/IV double KO strain (FcγRIII/IV$^{-/-}$) with a deletion of 29.1 kb between the two most distant loxP sites, respectively (Supplementary Figure 2A). The presence of the deletion was confirmed by a specific PCR and DNA sequencing (Supplementary Figure 2B). The absence of FcγRIII and FcγRIV on the surface of immune cells was confirmed by flow cytometry using FcγRIII and FcγRIV specific antibodies (data not shown). FcγRIII/IV$^{-/-}$ mice developed normally and showed normal breeding characteristics.

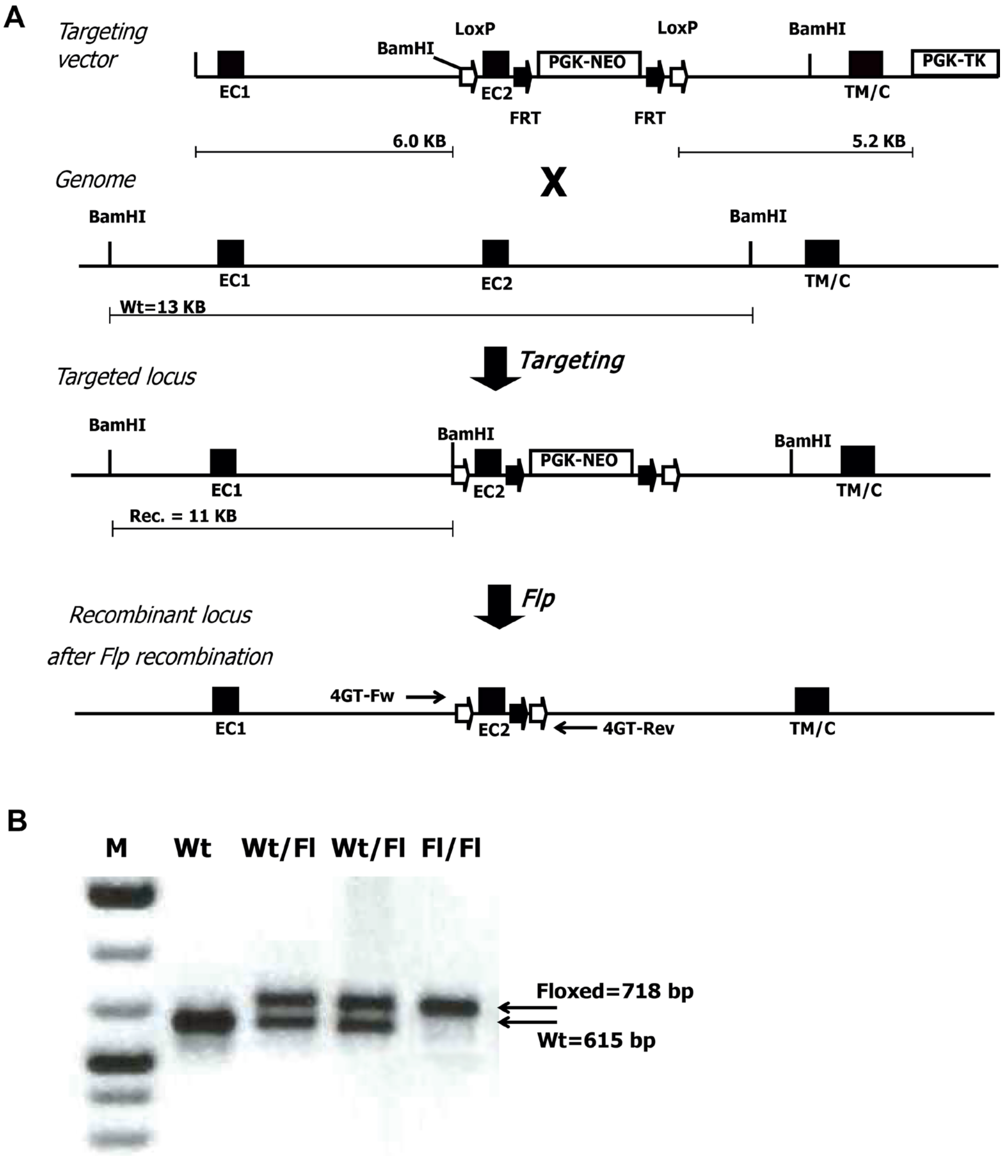

Supplementary Figure 1: Generation of a floxed FcγRIV allele by gene targeting in embryonic stem cells. (**A**) Exons are represented by closed rectangles. EC1 and EC2: extra cellular domain1 and 2 encoding exons; TM/C: transmembrane and cytoplasmic domain encoding exon. Open rectangles: selection marker genes required for gene targeting. (**B**) PCR analysis of genomic DNA from tail biopsies of offspring from an inter-cross of heterozygous floxed FcγRIV mice. Use of primer pair 4GT-Fw and 4GT-Rev results in the amplification of a 718 bp fragment of the floxed FcγRIV allele and the amplification of a 615 bp fragment of the wild-type (Wt) FcγRIV allele. Lane Fl/Fl: homozygous floxed FcγRIV mouse, lane Wt: Wt mouse. lanes Wt/Fl: heterozygous floxed FcγRIV mice. Primer sequences: 4GT-Fw: GGAGGCCAGAAGACTTTTA 4GT-Rev: GGAACTGTTTTGGAAGGATT.

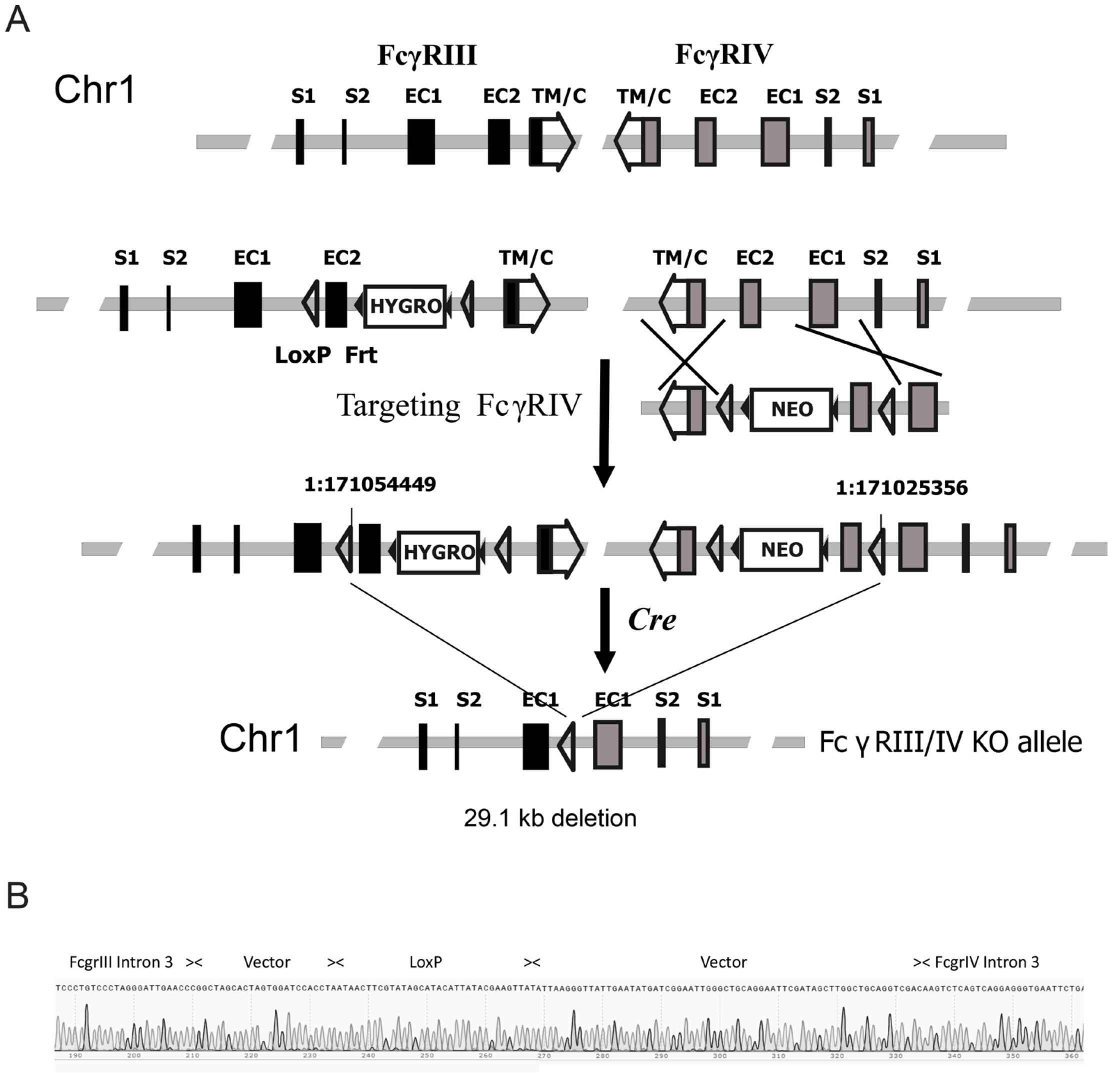

Supplementary Figure 2: Generation of the FcγRIII/IV$^{-/-}$ mouse model. (**A**) The FcγRIII/IVfl/fl mice were crossed with EIIaCre deleter mice and FcγRIII/IV KO offspring with a 29.093 bp deletion was selected. The locus is shown in reverse orientation in relation to the chromosomal nucleotide numbering. The exact location of the borders of the deletion (NC_000067.6:g.171054449_1:171025356del according to HGVS nomenclature) on chromosome 1 is depicted according to the mouse reference genome build GRCm38.p3 (C57BL/6J) provided by the Genome Reference Consortium. Exons are represented by closed rectangles. S1 and S2: signal peptide 1 and 2 encoding exons; EC1 and EC2: extra cellular domain1 and 2 encoding exons; TM/C: transmembrane and cytoplasmic domain encoding exon. Open rectangles: selection marker genes required for gene targeting. Top line: WT locus; second line: FcγRIII floxed locus; third line: FcγRIII/IV double floxed locus, bottom line: FcγRIII/IV$^{-/-}$ locus. (**B**) Core sequence of a unique 477 bp PCR fragment flanking the remaining LoxP site and bridging the 29.1 kb deletion in FcγRIII/IV$^{-/-}$ mice. Primer sequences: FcγRIII Geno Fw: GAGGGCATCCGATTTCATTA FcγRIV Null Fw TAGACTAAAGGTCATGTGTGATC
